# Book Review: American Trip: Set, Setting, and the Psychedelic Experience in the Twentieth Century

**DOI:** 10.3389/fpsyg.2021.732502

**Published:** 2021-08-02

**Authors:** Ephraim Shmaya Philip Lansky

**Affiliations:** Institute of Evolution, University of Haifa, Haifa, Israel

**Keywords:** consciousness, LSD, psilocybin, STS, context

## Summary

**Figure 1:**
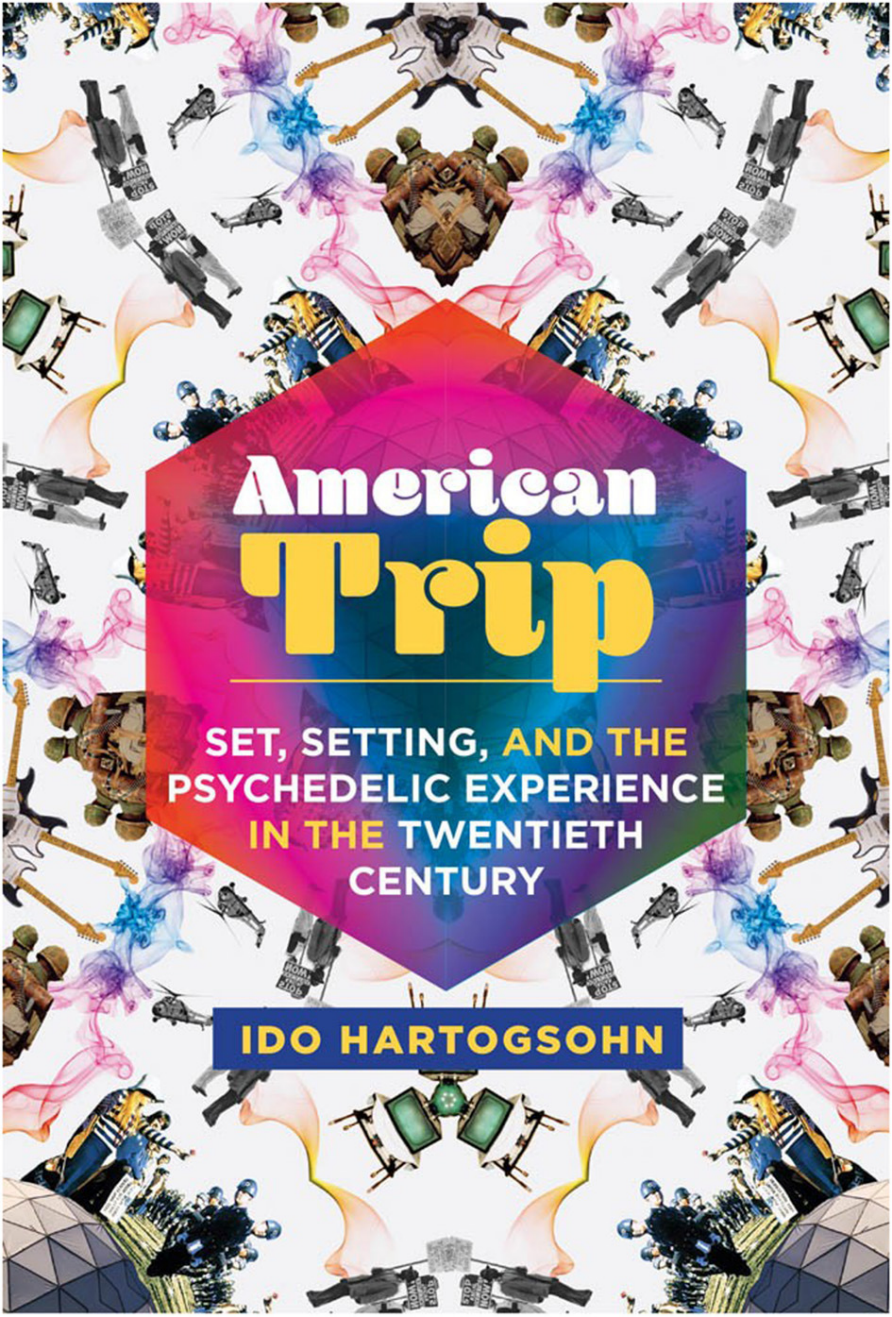
Front cover, of American Trip by Ido Hartogsohn, MIT Press, Cambridge, Massachusetts, 2020.

*American Trip* (Figure 1) is an erudite and readable social history of psychedelics in North America with special attention to *Set and Setting* as it shaped not only individual psychedelic experiences during the Sixties, but also the psychedelic experience of the society at large and its impact upon psychiatry, fashion, art, music, and the digital revolution. Hartogsohn elaborates how in the 1960's, America itself went on an epic acid trip.

In considering the book's chapters, the reader should remember that the social context for each chapter represents a different cultural and societal set and setting with its own specific effects on the LSD experiences of individuals taking LSD within that specific context. In this book review, “LSD” is used both for the specific compound, lysergic acid diethylamide, and to other psychedelic congeners (e.g., psilocybin, dimethyltryptamine) that act pharmacologically at serotonin receptors, especially 5-HT2AR, to mitigate serotonergic suppression of catecholaminergic (i.e., noradrenergic and dopaminergic) ergotropic arousal (Lansky, 1975), leading to rapture, ecstasy, introspection, synesthesia, hallucinations, telepathy, extracorporeal visitations, totem animals, past lives, death, birth, increased creativity, awareness, intensity, and “suchness” of the physical and spiritual worlds (Winkelman, 2021). The 10 chapters are:

1. The Experimental Psychosis Movement presents the *psychotomimetic*, prevailing paradigmatic model (Kuhn, 1962), that LSD effects mimicked those of natural psychoses, and so were a putative means for better understanding, and pharmacologically treating, psychoses. The movement was called Experimental Psychiatry.
2. LSD, the CIA, and the Military reveals the extent of the US Military's interest in LSD as a possible psychochemical weapon, and of the CIA's for intelligence gathering, all within the context of the Cold War and existential nuclear threats.
3. From Psychotomimetics to Psychedelics illustrates the shift from the psychotomimetic model of disease-mimicking, to the psychedelic model of healing from the psychedelic reset, especially under optimized set and setting of empathic therapists, comfortable physical space, and conducive music.
4. Experiments in Set and Setting follows Watts' maxim, “there is no drug reaction, but always setting-plus drug,” and that set may be optimized by pre-session rituals including meditation, introspection, fasting, and continence. In a classic study psychedelic inebriation was provided to divinity students in a chapel and was highly successful in producing mystical experience, while administration to prisoners in prison to reduce recidivism, was less effective, possibly due to the less benevolent setting.
5. Psychedelics, Creativity, and Culture explores LSD as a tool to enhance creativity in art and music, originally inspired by psychotomimetic research, it later came to mirror the spirit of creativity and boldness of the time, the can-do attitude, and the rise of space exploration, a metaphor for psychonauts.
6. Psychedelics Go to Silicon Valley describes the use of LSD to aid technical problem solving, and inspire design of personal computers and their software, with particular attention to the interface between computers, cybernetics, and psychedelic feedback loops.
7. The Psychedelic Controversy highlights the schism between cultural elements intent on demonizing LSD as a dangerous and subversive drug, and LSD adherents touting its catalytic guidance toward peace, love, harmony, and understanding.
8. American Trip pits the idea of *pharmacologicalism*, that drugs have inherent effects irrespective of set and setting, against the psychedelically inspired view that set and setting define drug experience of the drug's inherent properties.
9. LSD and the 1960s emphasizes the liberalisms of the period: sexual freedom, individuality, rebelliousness, and anti-psychiatry, coincident with material abundance and societal positive and adventurous spirit to the moon and beyond, and why these conditions supported the emergence of American psychedelic experimentation.
10. The Future of Set and Setting drives home the centrality of set and setting for defining drug effects. Much of the harm associated with drugs. especially LSD, was secondary to the societal settings at the time, and by easing restrictions, improving education, and bolstering public image of LSD, harm reduction can be more surely enhanced than by punishing users.

## Assessment

Overall, Hartogsohn successfully contained the paradox between pharmacologicalism on the one hand, and set and setting on the other as competing concepts within pharmacology for understanding drug effects. Pharmacological effects will always be important, but Hartogsohn's easy and informative style has done much good to bridge the gap between pharmacologicalism and the equally important extrapharmacological factors of set and setting, which are inclusive of societal as well as individual influences. *American Trip* is written from a sociological perspective, or that of the broader Science, Technology, and Society (STS), and will naturally appeal to social scientists or persons engaged with psychedelics, but the book is nonetheless also valuable for clinicians. Natural scientists pursuing deeper understanding of set and setting upon drug effects and experience will forgive Hartogsohn's occasional errors. For example, he called Ph.D. pharmacologist Roland Fischer a “psychotomimetic psychiatrist,” confused a strain of *Cannabis indica* with low THC and high CBD with a synthetic cannabinoid, and called quasi-psychedelic MDMA a “semi-psychedelic.” Nevertheless, as the mind provides cultural, personal, and transpersonal overlay to all degrees of physiological arousal (Fischer, 1971), so it is the set and setting, not the drug, that characterizes the individuality of drug experience. Hartogsohn's book is a seminal contribution to his discipline and the larger issue of extrapharmacological factors shaping drug effects.

## Implications

*American Trip* is relevant both on the societal and individual levels. Cultural context, laws, and mindset impact individual LSD experiences as do personal mindset and the physical setting of the inebriation. Pharmacologists informed by these findings will teach drug effects with latitude, varying according to the set and setting of the patient approaching, during, and following the session. Clinicians will ready their patients for LSD sessions through pre-session guidance, and the treatment room for the patient by attending to the patient's personal and transpersonal sensibilities. Therapeutic music should be selected according to the patient's input, musical sophistication, and cultural context. Set and setting will affect both the patient's reportage and the physical examination, and should be duly noted for each clinical encounter, prior to, during, following, and even excluding psychedelic interventions.

## Author Contributions

The author confirms being the sole contributor of this work and has approved it for publication.

## Conflict of Interest

The author declares that the research was conducted in the absence of any commercial or financial relationships that could be construed as a potential conflict of interest.

## Publisher's Note

All claims expressed in this article are solely those of the authors and do not necessarily represent those of their affiliated organizations, or those of the publisher, the editors and the reviewers. Any product that may be evaluated in this article, or claim that may be made by its manufacturer, is not guaranteed or endorsed by the publisher.
